# (1*R*,2*R*)-4-Benzoyl-2-benzo­yloxy-1-phenyl­butyl imidazole-1-carboxyl­ate

**DOI:** 10.1107/S1600536808004364

**Published:** 2008-02-22

**Authors:** David J. Fox, Sean Parris, Daniel Sejer Pedersen, Stuart Warren

**Affiliations:** a Department of Chemistry, University of Cambridge, Lensfield Road, Cambridge CB2 1EW, England

## Abstract

The title compound, C_28_H_24_N_2_O_5_, was prepared from (*E*)-2-cinnamyl-1,3-diphenyl­propane-1,3-dione using standard Sharpless asymmetric dihydroxy­lation conditions, followed by treatment with 1,1′-carbonyl diimidazole. In the crystal structure, the phenyl rings form inter­molecular face-to-face π–π contacts, with an inter­planar angle of 15.5 (2)° and a centroid–centroid distance of 4.73 (1) Å. One phenyl ring also forms a C—H⋯π contact to an adjacent imidazole ring, with an H⋯centroid distance of 3.18 Å.

## Related literature

For related literature, see: Fox *et al.* (2006 ▶); Kolb *et al.* (1994 ▶).
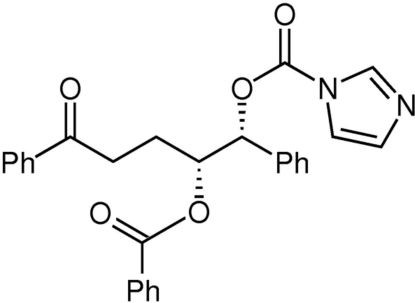


## Experimental

### 

#### Crystal data

C_28_H_24_N_2_O_5_
*M* *_r_* = 468.49
Orthorhombic, 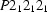

*a* = 5.9512 (2) Å
*b* = 18.1330 (7) Å
*c* = 22.4612 (12) Å
*V* = 2423.86 (18) Å^3^
*Z* = 4
Mo *K*α radiation
μ = 0.09 mm^−1^
*T* = 180 (2) K
0.37 × 0.05 × 0.02 mm

#### Data collection

Nonius KappaCCD diffractometer
Absorption correction: multi-scan (*SORTAV*; Blessing, 1995 ▶) *T*_min_ = 0.767, *T*_max_ = 0.998
7293 measured reflections
1416 independent reflections
1230 reflections with *I* > 2σ(*I*)
*R*_int_ = 0.076
θ_max_ = 20.4°

#### Refinement

*R*[*F* ^2^ > 2σ(*F* ^2^)] = 0.036
*wR*(*F* ^2^) = 0.080
*S* = 1.12
1416 reflections
317 parameters
H-atom parameters constrained
Δρ_max_ = 0.20 e Å^−3^
Δρ_min_ = −0.18 e Å^−3^

### 

Data collection: *COLLECT* (Nonius, 1998 ▶); cell refinement: *SCALEPACK* (Otwinowski & Minor, 1997 ▶); data reduction: *DENZO* (Otwinowski & Minor, 1997 ▶) and *SCALEPACK*; program(s) used to solve structure: *SIR92* (Altomare *et al.*, 1994 ▶); program(s) used to refine structure: *SHELXL97* (Sheldrick, 2008 ▶); molecular graphics: *SHELXTL* (Sheldrick, 2008 ▶); software used to prepare material for publication: *SHELXTL*.

## Supplementary Material

Crystal structure: contains datablocks global, I. DOI: 10.1107/S1600536808004364/pk2082sup1.cif

Structure factors: contains datablocks I. DOI: 10.1107/S1600536808004364/pk2082Isup2.hkl

Additional supplementary materials:  crystallographic information; 3D view; checkCIF report

## Figures and Tables

**Table 1:** Hydrogen-bond geometry (Å, °) *Cg*1 is the centroid of the imidazole ring.

*D*—H⋯*A*	*D*—H	H⋯*A*	*D*⋯*A*	*D*—H⋯*A*
C26—H26*A*⋯*Cg*1	0.95	3.18	4.07 (1)	155

**Fig. 1.**
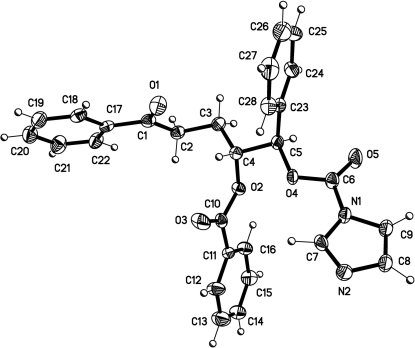
Molecular structure with displacement parameters drawn at the 30% probability level for non-H atoms.

**Fig. 2.**
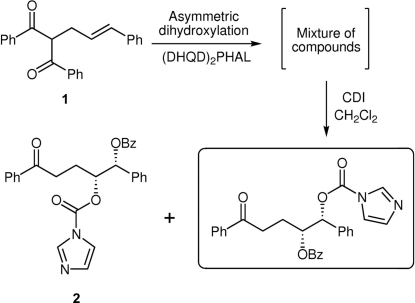
Summary of the synthetic procedure. CDI = 1,1'-carbonyldiimidazole, (DHQD)2PHAL = Hydroquinidine 1,4-phthalazinediyl diether.

## supplementary crystallographic information

### Comment

We recently reported a method for the preparation of optically active dihydrofurans starting from β-keto-diphenylphosphine oxides by intramolecular ring opening of cyclic carbonates (Fox *et al.*, 2006). Currently we are investigating this synthetic concept in more detail to include other anion-stabilizing groups. Seeking to extend the methodology to diketones we performed the Sharpless asymmetric dihydroxylation (Kolb *et al.*, 1994) on (*E*)-2-cinnamyl-1,3-diphenylpropane-1,3-dione, followed by treatment with 1,1'-carbonyl diimidazole. Surprisingly, this produced the title compound and the regioisomer (4*R*,5*R*)-5-benzoyloxy-1,5-diphenyl-4-imidazoyloxy-pentanone in a combined 50% yield. Presumably these products are formed by intramolecular acyl transfer during the asymmetric dihydroxylation step.

### Experimental

The synthetic procedure is summarized in Fig. 2. By the method of Sharpless and co-workers (Kolb *et al.*, 1994), olefin 1 (0.14 g, 0.41 mmol), was partially dissolved in *t*-BuOH (5 ml) with heating, and water (5 ml) was added. A freshly made mixture of OsCl_3_.xH_2_O (1 mol%), K_3_Fe(CN)_6_ (3 equiv.), K_2_CO_3_ (3 equiv.), MeSO_2_NH_2_ (1 equiv.) and hydroquinidine 1,4-phthalazinediyl diether (denoted (DHQD)_2_PHAL, 2 mol%) was added to the cooled solution (282 K) in one portion and it was stirred vigorously for 26 d. Sodium sulfite (*ca* 10 equiv.) was added and the reaction allowed to warm to room temperature with vigorous stirring. The reaction mixture was transferred to a separatory funnel and the organic layer separated, and concentrated in vacuo. The concentrated organic layer was partitioned between dichloromethane (20 ml) and water (20 ml), and the aqueous phase extracted with more dichloromethane (2 × 20 ml). The organic extracts were combined and washed with brine (20 ml), dried (Na_2_SO_4_), filtered and concentrated in vacuo. The residue was dissolved in anhydrous dichloromethane (5 ml) and 1,1'-carbonyldiimidazole (97 mg, 0.60 mmol) was added. After 7 h, the reaction was quenched with 1*M* aqueous hydrochloric acid (20 ml) and extracted with dichloromethane (3 × 25 ml). The combined organic phases were washed with saturated aqueous NaHCO_3_ (10 ml), dried (Na_2_SO_4_), filtered and concentrated *in vacuo*. The residue was purified by flash column chromatography (25–100% EtOAc in hexanes, *v*/*v*) to give a mixture of the title compound and the regioisomer 2 as a yellow oil (92 mg, 50%).

### Refinement

H atoms were placed geometrically and allowed to ride during refinement with C—H = 0.95–0.99 Å and with *U*_iso_(H) = 1.2*U*_eq_(C). Data were collected to θ = 20°, equivalent to a resolution of 1.04 Å. The resulting structure is therefore of relatively low precision. In the absence of significant anomalous scattering effects, 961 Friedel pairs were merged as equivalent data. The absolute structure is based on the known stereochemical outcome of the asymmetric dihydroxylation.

### Crystal data

**Table:**

C_28_H_24_N_2_O_5_	*F*_000_ = 984
*M**_r_* = 468.49	*D*_x_ = 1.284 Mg m^−^^3^
Orthorhombic, *P*2_1_2_1_2_1_	Mo *K*α radiation λ = 0.71073 Å
Hall symbol: P 2ac 2ab	Cell parameters from 7747 reflections
*a* = 5.9512 (2) Å	θ = 1.0–20.4º
*b* = 18.1330 (7) Å	µ = 0.09 mm^−^^1^
*c* = 22.4612 (12) Å	*T* = 180 (2) K
*V* = 2423.86 (18) Å^3^	Block, colourless
*Z* = 4	0.37 × 0.05 × 0.02 mm

### Data collection

**Table:**

Nonius KappaCCD diffractometer	1230 reflections with *I* > 2σ(*I*)
Radiation source: fine-focus sealed tube	*R*_int_ = 0.076
*T* = 180(2) K	θ_max_ = 20.4º
ω and φ scans	θ_min_ = 3.5º
Absorption correction: multi-scan(SORTAV; Blessing, 1995)	*h* = −5→5
*T*_min_ = 0.767, *T*_max_ = 0.998	*k* = −17→17
7293 measured reflections	*l* = −21→22
1416 independent reflections	

### Refinement

**Table:**

Refinement on *F*^2^	Hydrogen site location: inferred from neighbouring sites
Least-squares matrix: full	H-atom parameters constrained
*R*[*F*^2^ > 2σ(*F*^2^)] = 0.036	*w* = 1/[σ^2^(*F*_o_^2^) + (0.0295*P*)^2^ + 0.6591*P*] where *P* = (*F*_o_^2^ + 2*F*_c_^2^)/3
*wR*(*F*^2^) = 0.080	(Δ/σ)_max_ < 0.001
*S* = 1.12	Δρ_max_ = 0.20 e Å^−^^3^
1416 reflections	Δρ_min_ = −0.18 e Å^−^^3^
317 parameters	Extinction correction: SHELXL97 (Sheldrick, 2008), Fc^*^=kFc[1+0.001xFc^2^λ^3^/sin(2θ)]^-1/4^
Primary atom site location: structure-invariant direct methods	Extinction coefficient: 0.0071 (12)
Secondary atom site location: difference Fourier map	Absolute structure: In the absence of significant anomalous scattering effects, 961 Friedel pairs have been merged as equivalent data.

### Special details

**Table:**

Geometry. All e.s.d.'s (except the e.s.d. in the dihedral angle between two l.s. planes) are estimated using the full covariance matrix. The cell e.s.d.'s are taken into account individually in the estimation of e.s.d.'s in distances, angles and torsion angles; correlations between e.s.d.'s in cell parameters are only used when they are defined by crystal symmetry. An approximate (isotropic) treatment of cell e.s.d.'s is used for estimating e.s.d.'s involving l.s. planes.
Refinement. Refinement of *F*^2^ against ALL reflections. The weighted *R*-factor *wR* and goodness of fit *S* are based on *F*^2^, conventional *R*-factors *R* are based on *F*, with *F* set to zero for negative *F*^2^. The threshold expression of *F*^2^ > 2σ(*F*^2^) is used only for calculating *R*-factors(gt) *etc*. and is not relevant to the choice of reflections for refinement. *R*-factors based on *F*^2^ are statistically about twice as large as those based on *F*, and *R*- factors based on ALL data will be even larger.

### Fractional atomic coordinates and isotropic or equivalent isotropic displacement parameters (Å^2^)

**Table:**

	*x*	*y*	*z*	*U*_iso_*/*U*_eq_	
O1	0.4514 (6)	−0.04408 (18)	0.45568 (15)	0.0606 (10)	
O2	0.2125 (4)	0.04215 (14)	0.29114 (12)	0.0370 (7)	
O3	0.5837 (5)	0.06215 (19)	0.29436 (15)	0.0627 (10)	
O4	0.1450 (4)	0.17552 (15)	0.33765 (13)	0.0397 (8)	
O5	−0.1887 (6)	0.23422 (17)	0.33492 (17)	0.0663 (10)	
N1	0.1272 (6)	0.28828 (19)	0.29753 (17)	0.0397 (10)	
N2	0.4070 (6)	0.3576 (2)	0.26476 (18)	0.0477 (11)	
C1	0.4404 (8)	−0.0913 (3)	0.4173 (2)	0.0412 (12)	
C2	0.2591 (7)	−0.0889 (2)	0.3705 (2)	0.0411 (12)	
H2B	0.1717	−0.1353	0.3723	0.049*	
H2C	0.3303	−0.0861	0.3308	0.049*	
C3	0.0990 (7)	−0.0239 (2)	0.3779 (2)	0.0408 (12)	
H3A	−0.0419	−0.0347	0.3562	0.049*	
H3B	0.0611	−0.0186	0.4206	0.049*	
C4	0.1935 (7)	0.0484 (2)	0.35549 (18)	0.0338 (11)	
H4	0.3449	0.0574	0.3734	0.041*	
C5	0.0401 (7)	0.1133 (2)	0.3679 (2)	0.0367 (11)	
H5	−0.1116	0.1037	0.3503	0.044*	
C6	0.0092 (8)	0.2308 (3)	0.3247 (2)	0.0441 (12)	
C7	0.3534 (7)	0.2942 (3)	0.2878 (2)	0.0424 (12)	
H7	0.4588	0.2565	0.2968	0.051*	
C8	0.2059 (8)	0.3948 (3)	0.2583 (2)	0.0491 (13)	
H8	0.1919	0.4429	0.2419	0.059*	
C9	0.0327 (8)	0.3542 (3)	0.2781 (2)	0.0509 (13)	
H9	−0.1215	0.3677	0.2787	0.061*	
C10	0.4159 (8)	0.0525 (2)	0.2659 (2)	0.0393 (11)	
C11	0.4072 (7)	0.0490 (2)	0.20012 (19)	0.0348 (11)	
C12	0.5986 (8)	0.0679 (3)	0.1689 (2)	0.0578 (14)	
H12	0.7282	0.0843	0.1898	0.069*	
C13	0.6024 (9)	0.0632 (3)	0.1082 (3)	0.0734 (17)	
H13	0.7352	0.0757	0.0870	0.088*	
C14	0.4156 (10)	0.0405 (3)	0.0778 (2)	0.0636 (15)	
H14	0.4179	0.0385	0.0356	0.076*	
C15	0.2263 (9)	0.0207 (2)	0.1080 (2)	0.0547 (14)	
H15	0.0979	0.0038	0.0869	0.066*	
C16	0.2217 (7)	0.0254 (2)	0.1696 (2)	0.0441 (12)	
H16	0.0893	0.0121	0.1906	0.053*	
C17	0.6070 (8)	−0.1529 (2)	0.41677 (18)	0.0374 (11)	
C18	0.7758 (8)	−0.1535 (3)	0.4594 (2)	0.0466 (12)	
H18	0.7844	−0.1145	0.4875	0.056*	
C19	0.9307 (8)	−0.2095 (3)	0.4615 (3)	0.0601 (14)	
H19	1.0444	−0.2094	0.4912	0.072*	
C20	0.9211 (10)	−0.2657 (3)	0.4207 (3)	0.0641 (15)	
H20	1.0288	−0.3043	0.4220	0.077*	
C21	0.7573 (10)	−0.2660 (3)	0.3785 (2)	0.0646 (15)	
H21	0.7515	−0.3052	0.3504	0.077*	
C22	0.5981 (8)	−0.2100 (2)	0.3758 (2)	0.0513 (13)	
H22	0.4843	−0.2109	0.3462	0.062*	
C23	0.0174 (7)	0.1293 (2)	0.4332 (2)	0.0373 (12)	
C24	−0.1753 (8)	0.1095 (2)	0.4643 (3)	0.0546 (13)	
H24	−0.2968	0.0868	0.4439	0.066*	
C25	−0.1905 (11)	0.1227 (3)	0.5249 (3)	0.0722 (17)	
H25	−0.3217	0.1085	0.5461	0.087*	
C26	−0.0174 (14)	0.1563 (4)	0.5544 (3)	0.080 (2)	
H26	−0.0293	0.1653	0.5959	0.097*	
C27	0.1732 (11)	0.1770 (3)	0.5245 (3)	0.0692 (16)	
H27	0.2924	0.2006	0.5451	0.083*	
C28	0.1910 (8)	0.1634 (2)	0.4642 (2)	0.0498 (13)	
H28	0.3237	0.1775	0.4436	0.060*	

### Atomic displacement parameters (Å^2^)

**Table:**

	*U*^11^	*U*^22^	*U*^33^	*U*^12^	*U*^13^	*U*^23^
O1	0.077 (2)	0.052 (2)	0.052 (2)	0.0064 (19)	−0.0150 (19)	−0.015 (2)
O2	0.0302 (18)	0.0434 (17)	0.0374 (19)	−0.0031 (14)	−0.0014 (15)	−0.0026 (15)
O3	0.0305 (18)	0.104 (3)	0.053 (2)	−0.0117 (19)	−0.0072 (19)	−0.005 (2)
O4	0.0301 (16)	0.0336 (17)	0.055 (2)	0.0057 (15)	−0.0016 (15)	0.0084 (16)
O5	0.034 (2)	0.058 (2)	0.107 (3)	0.0085 (17)	0.001 (2)	0.021 (2)
N1	0.034 (2)	0.036 (2)	0.049 (2)	0.003 (2)	−0.0054 (19)	0.006 (2)
N2	0.043 (3)	0.047 (3)	0.053 (3)	−0.002 (2)	0.001 (2)	0.004 (2)
C1	0.051 (3)	0.033 (3)	0.039 (3)	−0.005 (3)	0.005 (3)	−0.001 (3)
C2	0.046 (3)	0.035 (2)	0.042 (3)	−0.004 (2)	0.000 (3)	0.002 (2)
C3	0.042 (3)	0.039 (3)	0.041 (3)	−0.002 (2)	−0.003 (2)	0.003 (2)
C4	0.031 (2)	0.039 (3)	0.031 (3)	−0.001 (2)	−0.002 (2)	−0.002 (2)
C5	0.027 (2)	0.036 (3)	0.046 (3)	−0.002 (2)	−0.004 (2)	0.006 (2)
C6	0.034 (3)	0.042 (3)	0.056 (3)	0.002 (3)	−0.005 (2)	0.002 (3)
C7	0.030 (3)	0.052 (3)	0.046 (3)	0.008 (2)	−0.004 (2)	−0.001 (3)
C8	0.044 (3)	0.040 (3)	0.064 (4)	0.006 (3)	−0.002 (3)	0.008 (3)
C9	0.044 (3)	0.043 (3)	0.066 (4)	0.008 (3)	0.000 (3)	0.011 (3)
C10	0.028 (3)	0.034 (3)	0.056 (3)	−0.002 (2)	0.000 (3)	0.003 (2)
C11	0.035 (3)	0.035 (2)	0.034 (3)	0.001 (2)	0.003 (3)	0.004 (2)
C12	0.043 (3)	0.085 (4)	0.045 (4)	−0.013 (3)	0.001 (3)	0.010 (3)
C13	0.049 (4)	0.113 (5)	0.059 (4)	−0.003 (3)	0.006 (3)	0.020 (4)
C14	0.066 (4)	0.079 (4)	0.047 (4)	0.007 (3)	0.014 (4)	0.007 (3)
C15	0.060 (4)	0.054 (3)	0.050 (4)	−0.008 (3)	0.000 (3)	−0.008 (3)
C16	0.040 (3)	0.047 (3)	0.046 (3)	−0.011 (2)	0.003 (3)	−0.004 (2)
C17	0.050 (3)	0.035 (3)	0.028 (3)	−0.004 (2)	0.002 (2)	0.006 (2)
C18	0.051 (3)	0.046 (3)	0.043 (3)	−0.011 (3)	−0.002 (3)	0.010 (2)
C19	0.051 (3)	0.062 (4)	0.067 (4)	−0.001 (3)	−0.008 (3)	0.018 (3)
C20	0.062 (4)	0.055 (4)	0.075 (4)	0.009 (3)	−0.002 (4)	0.011 (3)
C21	0.085 (4)	0.043 (3)	0.066 (4)	0.009 (3)	−0.004 (4)	−0.006 (3)
C22	0.063 (3)	0.042 (3)	0.049 (3)	0.005 (3)	−0.006 (3)	0.005 (3)
C23	0.035 (3)	0.035 (3)	0.042 (3)	0.005 (2)	−0.001 (2)	0.001 (2)
C24	0.050 (3)	0.052 (3)	0.062 (4)	0.008 (3)	0.010 (3)	−0.001 (3)
C25	0.091 (5)	0.064 (4)	0.062 (5)	0.017 (4)	0.034 (4)	0.007 (3)
C26	0.122 (6)	0.072 (4)	0.047 (4)	0.030 (4)	−0.001 (5)	−0.012 (4)
C27	0.083 (4)	0.064 (4)	0.061 (5)	0.012 (3)	−0.017 (4)	−0.021 (3)
C28	0.053 (3)	0.048 (3)	0.049 (4)	0.004 (3)	−0.008 (3)	−0.006 (3)

### Geometric parameters (Å, °)

**Table:**

O1—C1	1.217 (5)	C12—C13	1.368 (7)
O2—C10	1.350 (5)	C12—H12	0.950
O2—C4	1.454 (5)	C13—C14	1.367 (7)
O3—C10	1.199 (5)	C13—H13	0.950
O4—C6	1.321 (5)	C14—C15	1.363 (7)
O4—C5	1.458 (5)	C14—H14	0.950
O5—C6	1.201 (5)	C15—C16	1.386 (6)
N1—C7	1.368 (5)	C15—H15	0.950
N1—C9	1.392 (5)	C16—H16	0.950
N1—C6	1.397 (5)	C17—C22	1.386 (6)
N2—C7	1.300 (5)	C17—C18	1.387 (6)
N2—C8	1.382 (5)	C18—C19	1.373 (6)
C1—C17	1.494 (6)	C18—H18	0.950
C1—C2	1.506 (6)	C19—C20	1.370 (7)
C2—C3	1.526 (5)	C19—H19	0.950
C2—H2B	0.990	C20—C21	1.360 (7)
C2—H2C	0.990	C20—H20	0.950
C3—C4	1.512 (5)	C21—C22	1.390 (6)
C3—H3A	0.990	C21—H21	0.950
C3—H3B	0.990	C22—H22	0.950
C4—C5	1.515 (5)	C23—C24	1.390 (6)
C4—H4	1.000	C23—C28	1.391 (6)
C5—C23	1.501 (6)	C24—C25	1.386 (8)
C5—H5	1.000	C24—H24	0.950
C7—H7	0.950	C25—C26	1.367 (8)
C8—C9	1.342 (6)	C25—H25	0.950
C8—H8	0.950	C26—C27	1.371 (8)
C9—H9	0.950	C26—H26	0.950
C10—C11	1.479 (6)	C27—C28	1.379 (7)
C11—C16	1.368 (6)	C27—H27	0.950
C11—C12	1.381 (6)	C28—H28	0.950
			
C10—O2—C4	118.5 (3)	C13—C12—C11	120.3 (5)
C6—O4—C5	115.4 (3)	C13—C12—H12	119.9
C7—N1—C9	106.2 (4)	C11—C12—H12	119.9
C7—N1—C6	128.6 (4)	C14—C13—C12	120.2 (5)
C9—N1—C6	125.1 (4)	C14—C13—H13	119.9
C7—N2—C8	105.1 (4)	C12—C13—H13	119.9
O1—C1—C17	119.7 (4)	C15—C14—C13	120.2 (5)
O1—C1—C2	120.8 (4)	C15—C14—H14	119.9
C17—C1—C2	119.4 (4)	C13—C14—H14	119.9
C1—C2—C3	113.2 (3)	C14—C15—C16	119.8 (5)
C1—C2—H2B	108.9	C14—C15—H15	120.1
C3—C2—H2B	108.9	C16—C15—H15	120.1
C1—C2—H2C	108.9	C11—C16—C15	120.3 (4)
C3—C2—H2C	108.9	C11—C16—H16	119.9
H2B—C2—H2C	107.7	C15—C16—H16	119.9
C4—C3—C2	113.7 (3)	C22—C17—C18	118.6 (4)
C4—C3—H3A	108.8	C22—C17—C1	122.6 (4)
C2—C3—H3A	108.8	C18—C17—C1	118.8 (4)
C4—C3—H3B	108.8	C19—C18—C17	121.1 (5)
C2—C3—H3B	108.8	C19—C18—H18	119.5
H3A—C3—H3B	107.7	C17—C18—H18	119.5
O2—C4—C3	107.0 (3)	C20—C19—C18	119.9 (5)
O2—C4—C5	106.9 (3)	C20—C19—H19	120.1
C3—C4—C5	112.8 (3)	C18—C19—H19	120.1
O2—C4—H4	110.0	C21—C20—C19	120.0 (5)
C3—C4—H4	110.0	C21—C20—H20	120.0
C5—C4—H4	110.0	C19—C20—H20	120.0
O4—C5—C23	110.1 (3)	C20—C21—C22	121.0 (5)
O4—C5—C4	104.9 (3)	C20—C21—H21	119.5
C23—C5—C4	112.6 (3)	C22—C21—H21	119.5
O4—C5—H5	109.7	C17—C22—C21	119.4 (4)
C23—C5—H5	109.7	C17—C22—H22	120.3
C4—C5—H5	109.7	C21—C22—H22	120.3
O5—C6—O4	126.6 (4)	C24—C23—C28	118.4 (4)
O5—C6—N1	122.6 (4)	C24—C23—C5	121.0 (4)
O4—C6—N1	110.8 (4)	C28—C23—C5	120.6 (4)
N2—C7—N1	112.0 (4)	C25—C24—C23	120.2 (5)
N2—C7—H7	124.0	C25—C24—H24	119.9
N1—C7—H7	124.0	C23—C24—H24	119.9
C9—C8—N2	111.3 (4)	C26—C25—C24	120.2 (5)
C9—C8—H8	124.4	C26—C25—H25	119.9
N2—C8—H8	124.4	C24—C25—H25	119.9
C8—C9—N1	105.4 (4)	C25—C26—C27	120.5 (5)
C8—C9—H9	127.3	C25—C26—H26	119.7
N1—C9—H9	127.3	C27—C26—H26	119.7
O3—C10—O2	122.9 (4)	C26—C27—C28	119.7 (5)
O3—C10—C11	124.7 (4)	C26—C27—H27	120.1
O2—C10—C11	112.5 (4)	C28—C27—H27	120.1
C16—C11—C12	119.3 (4)	C27—C28—C23	120.9 (5)
C16—C11—C10	122.9 (4)	C27—C28—H28	119.5
C12—C11—C10	117.8 (4)	C23—C28—H28	119.5
			
O1—C1—C2—C3	−0.8 (6)	C16—C11—C12—C13	0.1 (7)
C17—C1—C2—C3	178.6 (4)	C10—C11—C12—C13	177.7 (5)
C1—C2—C3—C4	78.5 (4)	C11—C12—C13—C14	0.8 (8)
C10—O2—C4—C3	−122.7 (4)	C12—C13—C14—C15	−1.6 (9)
C10—O2—C4—C5	116.2 (4)	C13—C14—C15—C16	1.5 (8)
C2—C3—C4—O2	67.6 (4)	C12—C11—C16—C15	−0.1 (7)
C2—C3—C4—C5	−175.2 (4)	C10—C11—C16—C15	−177.6 (4)
C6—O4—C5—C23	−80.9 (4)	C14—C15—C16—C11	−0.7 (7)
C6—O4—C5—C4	157.7 (3)	O1—C1—C17—C22	177.3 (4)
O2—C4—C5—O4	−56.5 (4)	C2—C1—C17—C22	−2.1 (6)
C3—C4—C5—O4	−173.8 (3)	O1—C1—C17—C18	−2.0 (6)
O2—C4—C5—C23	−176.2 (3)	C2—C1—C17—C18	178.6 (4)
C3—C4—C5—C23	66.5 (4)	C22—C17—C18—C19	−0.5 (6)
C5—O4—C6—O5	−0.5 (7)	C1—C17—C18—C19	178.8 (4)
C5—O4—C6—N1	178.2 (3)	C17—C18—C19—C20	0.7 (7)
C7—N1—C6—O5	174.1 (5)	C18—C19—C20—C21	−0.5 (8)
C9—N1—C6—O5	−1.6 (8)	C19—C20—C21—C22	0.1 (8)
C7—N1—C6—O4	−4.7 (7)	C18—C17—C22—C21	0.1 (6)
C9—N1—C6—O4	179.6 (4)	C1—C17—C22—C21	−179.2 (4)
C8—N2—C7—N1	−0.9 (5)	C20—C21—C22—C17	0.1 (7)
C9—N1—C7—N2	0.6 (6)	O4—C5—C23—C24	139.4 (4)
C6—N1—C7—N2	−175.8 (4)	C4—C5—C23—C24	−103.9 (4)
C7—N2—C8—C9	0.9 (5)	O4—C5—C23—C28	−41.8 (5)
N2—C8—C9—N1	−0.5 (5)	C4—C5—C23—C28	74.9 (5)
C7—N1—C9—C8	0.0 (5)	C28—C23—C24—C25	−0.8 (7)
C6—N1—C9—C8	176.5 (4)	C5—C23—C24—C25	178.0 (4)
C4—O2—C10—O3	4.6 (6)	C23—C24—C25—C26	0.8 (7)
C4—O2—C10—C11	−176.5 (3)	C24—C25—C26—C27	−0.2 (8)
O3—C10—C11—C16	168.4 (4)	C25—C26—C27—C28	−0.5 (8)
O2—C10—C11—C16	−10.6 (6)	C26—C27—C28—C23	0.5 (7)
O3—C10—C11—C12	−9.1 (7)	C24—C23—C28—C27	0.2 (7)
O2—C10—C11—C12	172.0 (4)	C5—C23—C28—C27	−178.7 (4)

### Hydrogen-bond geometry (Å, °)

**Table:**

*D*—H···*A*	*D*—H	H···*A*	*D*···*A*	*D*—H···*A*
C26—H26A···Cg1	0.95	3.18	4.07 (1)	155
